# Patterns of activity correlate with symptom severity in major depressive disorder patients

**DOI:** 10.1038/s41398-022-01989-9

**Published:** 2022-06-02

**Authors:** S. Spulber, F. Elberling, J. Svensson, M. Tiger, S. Ceccatelli, J. Lundberg

**Affiliations:** 1 Department of Neuroscience, Karolinska Institutet, Stockholm, Sweden; 2 Department of Clinical Neuroscience, Centre for Psychiatry Research, Karolinska Institutet & Stockholm Health Care Services, Region Stockholm, Karolinska University Hospital, Stockholm, Sweden

**Keywords:** Depression, Predictive markers

## Abstract

Objective measures, such as activity monitoring, can potentially complement clinical assessment for psychiatric patients. Alterations in rest–activity patterns are commonly encountered in patients with major depressive disorder. The aim of this study was to investigate whether features of activity patterns correlate with severity of depression symptoms (evaluated by Montgomery–Åsberg Rating Scale (MADRS) for depression). We used actigraphy recordings collected during ongoing major depressive episodes from patients not undergoing any antidepressant treatment. The recordings were acquired from two independent studies using different actigraphy systems. Data was quality-controlled and pre-processed for feature extraction following uniform procedures. We trained multiple regression models to predict MADRS score from features of activity patterns using brute-force and semi-supervised machine learning algorithms. The models were filtered based on the precision and the accuracy of fitting on training dataset before undergoing external validation on an independent dataset. The features enriched in the models surviving external validation point to high depressive symptom severity being associated with less complex activity patterns and stronger coupling to external circadian entrainers. Our results bring proof-of-concept evidence that activity patterns correlate with severity of depressive symptoms and suggest that actigraphy recordings may be a useful tool for individual evaluation of patients with major depressive disorder.

## Introduction

Wrist actigraphy is a technique that allows long-term recording of activity with minimal discomfort and safety challenges for the subject. The availability of off-the-shelf medical or research grade actigraphy devices has enabled larger scale collection of actigraphy data [1], and there is increasing consensus around feature engineering and biological interpretations of specific parameters derived from analysis of actigraphic recordings [2–4]. Analyses of rest and activity patterns have highlighted specific alterations in psychiatric disorders as compared to healthy controls, as well as among different disorders [5–8]. Patients suffering from an episode of major depressive disorder (MDD) display globally lower levels of activity [9], with shorter diurnal activity period and shorter bouts of activity [6, 10], and flattened circadian fluctuations in activity levels [1, 11–13]. In addition, symptom severity has been shown to correlate with the amount of moderate intensity physical activity [14] and with the number of sedentary bouts [15], while increasing the level of activity by structured, supervised physical activity has been proven to be an effective antidepressant intervention [16–18].

The mechanisms regulating circadian patterns of activity involve complex interactions between environmental cues (i.e., light–dark cycle, social interactions, meals, and physical activity) and the internal clock (located in the suprachiasmatic nucleus of the anterior hypothalamus). It has been shown that gene level alterations intrinsic to the molecular clock mechanism (e.g., strength of coupling in circadian oscillations in clock gene expression) are associated with depression [19–21]. In addition, weaker coupling between the central clock and peripheral oscillators has been demonstrated in depressed patients and suicide victims [22], and has been verified in experimental models of depression [23, 24]. However, correlations between activity patterns and symptom severity in depression have hitherto received very little attention.

The aim of his paper was to investigate whether features of activity patterns from actigraphic recordings correlate with the severity of depression symptoms (estimated using the interview-based Montgomery–Åsberg Rating Scale (MADRS) for depression [25]) in adult patients with major depressive disorder before treatment. To this end we have analyzed actigraphy data using a battery of non-parametric and non-linear approaches for feature extraction. We then trained and validated linear models to predict symptom severity using the extracted features. Our data provide proof-of-concept support for correlation between symptom severity and activity patterns for patients with ongoing major depression episode. Lastly, we discuss the biological significance of the features with highest leverage in the models.

## Materials and methods

### Actigraphy data collection

Actigraphy data was collected in clinical trials approved by the Swedish Research Ethics Committee (Dnr. 2017/799-31; Dnr. 2014/452-31). All subjects provided informed consent before data collection. The training dataset was collected as part of the previously published study on serotonin transporter availability in patients given cognitive behavioral therapy (CBT) for the treatment of a major depressive episode [26]. Briefly, 17 subjects were recruited by advertisements in local newspapers. Diagnosis of depression was established after full psychiatric assessment by a psychiatrist or by resident physician supervised by a senior psychiatrist. The study included patients with an ongoing major depressive episode according to DSM-IV criteria, with a history of at least one prior episode and that were not undergoing any psychopharmacological treatment for MDD. The included patients had a MADRS score between 18 and 35. Activity was recorded in 12 subjects for at least 7 consecutive days immediately prior to starting the CBT treatment program. Actigraphic recordings were acquired using GENEActiv Original wrist-worn actigraphs (Activinsights, Cambs, UK). The devices use three-dimensional accelerometers (up to 8×*g*; resolution of 3.9 m*g*) at 30 Hz sampling rate to record wrist movement. The patients were instructed to wear the actigraph continuously on the wrist of the non-dominant arm and not remove it unless for personal safety reasons (e.g., sauna, or contact sports such as martial arts, rock climbing, or volleyball). The raw data was downloaded using proprietary software, then processed in Matlab (The Mathworks, Natick, MD, USA), using a modified version of the code (https://github.com/DavidRConnell/geneactivReader), as described earlier [27]. Briefly, the Euclidean norm of change in acceleration vector was first smoothed using a rolling Gauss window spanning 30 consecutive datapoints (1 s), then a high-pass filter was applied (threshold: 20 m*g* = 196 mm/s^2^) before computing the sum of changes in acceleration vectors over 1 min epochs (1440 samples/24 h). A total of 12 recordings spanning between 6 and 12 consecutive days were included in the training dataset.

The external validation was performed on an independent dataset. The test dataset consisted of actigraphy data recorded as part of a clinical study addressing the effects of ketamine on serotonin receptor binding in patients with treatment-resistant depression [28]. Briefly, the study included 39 patients with an ongoing major depressive episode, with MADRS ≥ 20, resistant to selective serotonin reuptake inhibitor (SSRI) treatment in an adequate dose for at least 4 weeks. Ongoing antidepressant treatment was discontinued and actigraphy data was collected after a washout period of at least 5 times the half-life of the SSRI. The patients were instructed to wear the actigraph continuously on the wrist of the non-dominant arm and not remove it unless for personal safety reasons. The recording started prior to the first ketamine infusion and continued for the duration of the ketamine treatment program. For the purpose of this study, we cropped the recordings to include the period immediately prior to the first ketamine infusion (i.e., after drug washout period). Actigraphic recordings were acquired using Actiwatch 2 wrist-worn devices (Philips Respironics, Murrysville, PA, USA) set to record activity only integrated over 1 min epochs. The raw data was downloaded according to manufacturer’s instructions (Actiware 6.0.9, Philips Respironics) then exported as text files. The text file was imported to Matlab™ using a custom function designed to yield an output similar to the one generated by the import function for GENEActiv devices. A total of 23 recordings spanning between 2 and 7 consecutive days were included in the test dataset.

### Quality control and inclusion criteria

**Fig. 1:**
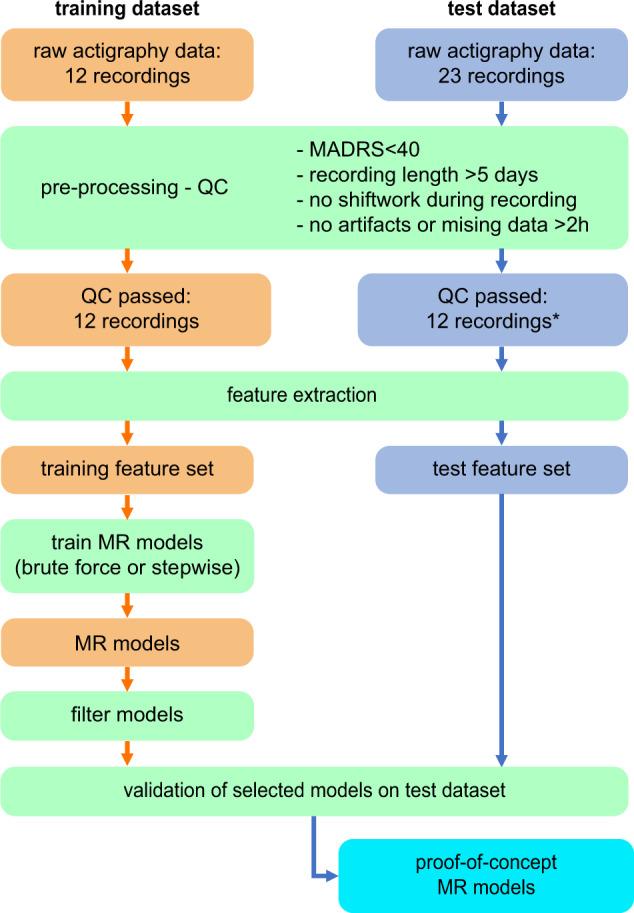
Workflow for data analysis. All recordings were screened by the same investigator, blind torecording conditions. MR - multiple regression; * - see main text for details on reasons to not pass QC.

The recordings in both train and test dataset underwent the same screening procedure and were assessed by the same observer blind to the recording conditions. All recordings were first inspected visually using a standardized procedure designed to identify stretches of missing data, artifacts, and gross abnormal circadian patterns of activity (e.g., shift-work). Intervals containing suspected shift-work (not reported at the time of recording), suspected artifacts, or missing data, were cropped out. Individual cropped recordings were included if they fulfilled the following requirements: mild to moderate symptom severity (recordings from patients with MADRS > 40 were not included, given the MADRS range was limited to 35 in the training dataset); minimum recording length 5 days; the recording did not include shift-work periods or other exceptional events with potentially high impact on the subject’s circadian patterns of activity (as identified on the actigraphy recording); the recording was continuous and did not include stretches of missing data longer than 2 h (e.g., due to not wearing the recording device for personal safety reasons). In the train dataset, all recordings passed the quality control procedure. In the test dataset, recordings were rejecting for the following reasons: MADRS > 40 (1); recording length <5 consecutive days (7); shift-work during recording time (2); missing data (2). This yielded a total of 24 recordings to use for further analyses: 12 for train and 12 for test datasets (see also Fig. 1 for description of workflow). All actigraphy recording originated from different patients (i.e., no patient provided more than 1 recording).

### Pre-processing and feature extraction

**Fig. 2:**
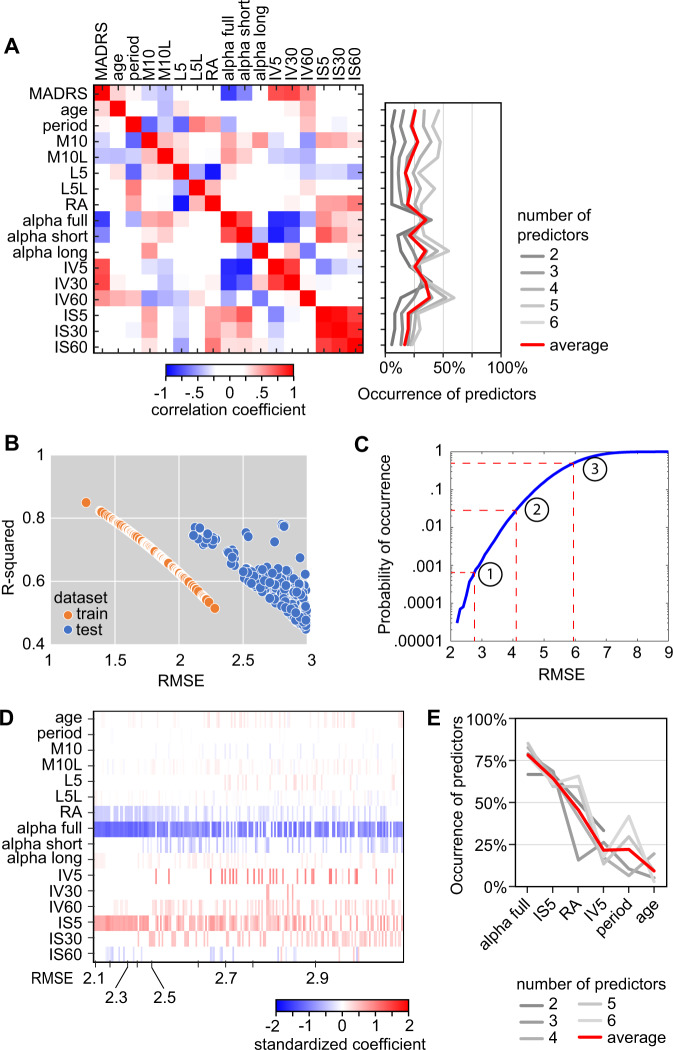
Development of multiple regression models by brute force. **A** Correlation matrix for all predictors used for model training (training dataset only). The occurrence of individual predictors in the models surviving internal validation is depicted in the right panel. **B** Performance of models surviving external validation (*N* = 192) on train and test (independent) datasets. **C** Cumulative histogram of accuracy (RMSE) achieved by 1 million simulated random datasets with overlayed reference values: (1) average RMSE for models surviving external validation; (2) accuracy level of dummy model; (3) average accuracy of simulated datasets. **D** Standardized coefficients for all models surviving external validation criteria (*N* = 192), sorted by RMSE on test dataset in ascending order. Each model is depicted by one column and corresponds to a point in the scatterplot in (**B**). **E** Occurrence of most frequently encountered individual predictors the models surviving external validation criteria. “period” and “age” are included based on potential biological relevance.

All data processing was performed in Matlab using custom implementations of publicly available algorithms. The selection of features took into account the fact that the training and test datasets were acquired using different recording devices and required different pre-processing steps. Therefore, we aimed to include primarily features independent from the magnitude of the reported activity (average hourly activity level during the most active 10 consecutive hours and least active 5 consecutive hours—M10 and L5, respectively—depend on the output magnitude) and focused on features describing the regularity, fragmentation, and complexity of circadian patterns of activity. Feature extraction was performed on recordings cropped between first and last midnight to yield an integer number of 24 h periods. The following features were extracted: circadian period; scaling exponent [4]; intradaily variability; interdaily stability; circadian peak and trough; relative amplitude [1, 2, 27]. The features extracted and included as predictors for model development are listed in Table 1, and the correlation matrix for all predictors as well as outcome variable (MADRS) is shown in Fig. 2A.

**Table 1:** Features extracted and comparison between datasets.

Train dataset (*N* = 12)						Test dataset (*N* = 12)			
Average		SD	Min	Max	Average	SD	min	max	*p*
MADRS	29.2	3.3	23	35	28.8	4.2	24	37	0.83
Rec. length	9.9	2.5	6	13	6.7	0.8	5	7	*0.00*
Age	48.1	14.7	25	78	42.1	12.1	20	63	0.29
Sex (M/F)		3/9				5/7			0.39
Period	24.0	0.1	23.9	24.2	24.1	0.6	23.5	25.4	0.53
M10*	9511	2771	4064	13,058	337	126	152	656	*0.00*
M10L	14.9	2.6	11.8	18.9	15.8	2.2	12.4	20.4	0.36
L5*	728	513	255	1673	25	16	8	62	*0.00*
L5L	3.8	1.0	1.6	5.7	5.3	3.3	0.9	11.2	0.15
RA	0.86	0.08	0.69	0.94	0.85	0.09	0.65	0.96	0.82
Alpha full	0.99	0.03	0.95	1.05	0.99	0.02	0.95	1.03	0.95
Alpha short	1.01	0.04	0.95	1.10	0.98	0.03	0.93	1.03	0.08
Alpha long	1.03	0.08	0.91	1.19	0.99	0.32	0.00	1.22	0.65
IV5	0.51	0.09	0.35	0.68	0.51	0.05	0.42	0.60	0.80
IV30	0.70	0.15	0.47	1.02	0.70	0.13	0.52	1.00	0.99
IV60	0.83	0.13	0.64	1.12	0.73	0.14	0.43	0.95	0.08
IS5	0.33	0.04	0.29	0.41	0.31	0.08	0.19	0.44	0.55
IS30	0.39	0.04	0.33	0.45	0.39	0.11	0.22	0.56	0.96
IS60	0.51	0.06	0.38	0.62	0.45	0.14	0.25	0.63	0.15

Asterisks indicate features dependent on data collection device, for which the magnitude depends on the output of specific sensors. *p*-values are calculated using Student’s *t*-test (two-sided) for continuous variables, or chi-square for ratios as appropriate.

Circadian period was estimated using the Lomb–Scargle algorithm optimized for Matlab implementation [29]. The Lomb–Scargle periodogram was preferred over the most commonly used Sokolove–Bushell [30] algorithm because the latter has been shown to yield period estimates biased towards periods below 24 h [31]. The circadian period was calculated over the entire recording using an oversampling factor of 10 to yield a minute-range resolution of the estimate. The scaling exponent for detrended fluctuation analysis was calculated for the magnitude of measured activity in 1-min bins using boxes equally spaced on a logarithmic scale between 4 min (4 consecutive samples) and 24 h (1440 consecutive samples) as described by Hu et al. [4]. The scaling exponent is a feature of the intrinsic regulatory mechanisms controlling the rest/activity patterns. It has not been shown to be sensitive to extrinsic factors the subject is exposed to in normal daily activity, but is altered as a result of disease [4, 6, 7]. Intradaily variability estimate the fragmentation of activity patterns by calculating the ratio between mean squared differences between consecutive intervals and the mean squared difference from global mean activity per interval; it increases as the frequency and the magnitude of transitions between rest and active intervals increase, and decreases as the active and inactive intervals consolidate [2]. Interdaily stability evaluates the coupling between activity patterns and circadian entrainers as the ratio between variability around circadian profile and global variability. High values indicate consistent activity patterns across days, consistent with strong coupling between activity and circadian entrainers. The relative amplitude of circadian rhythms of activity (RA) estimates the robustness of average circadian rhythms [1, 2]. The range of RA is bounded between 0 (no circadian rhythms) and 1 (robust circadian rhythms, with consistent timing of consolidated rest interval >5 h across days).

### Model development and validation

To limit the risk of overfitting, the maximum number of predictors was limited to 6, which corresponds to a ratio of minimum 2 subjects/predictor [32]. First, we used a brute force method to explore all models possible in the given feature space. Models based on 1–6 predictors were generated using all combinations possible in the feature space. Second, we refined the procedure of generation and selection of models by using machine learning (ML) algorithms to train models of increasing complexity (forward stepwise multiple regression). We started by using the entire feature space, then we manually restricted the inclusion of features such as subject age and circadian period in the model before running the ML algorithm again. We iterated the entire procedure using *F* statistic or Akaike information criterion (AIC) as criterion for inclusion of predictors in the model.

Next, the models were filtered using the following criteria: variable inflation factor (VIF) < 5 for any single predictor; coefficient of determination (*R*-squared) > 0.5; and root-mean-square error (RMSE) < 3. The models surviving filtering were then used for assessing the occurrence of individual predictors.

We then performed an external validation of filtered models. To this end we evaluated the performance of models validated as described above on an independent (test) dataset. The performance of individual models was assessed using the coefficient of determination (R-squared), and the RMSE for predicted vs. observed MADRS to evaluate the precision and the accuracy of the estimate (Fig. 2B). We filtered the models to be further analyzed as follows: significant correlation between predicted and observed MADRS (*p* < 0.05 corresponding to Pearson *R* > 0.576) and RMSE < 3 for test dataset.

To provide an internal reference for model performance, we generated a dummy model (predicted score = average score for the test dataset), and a random prediction dataset (1 million simulated sets of random integer values in the same range as the test dataset). The probability distribution of prediction accuracy (RMSE) is depicted in Fig. 2C.

The frequency of occurrence for individual predictors in validated models was calculated as the number of models including each unique predictor divided by the total number of validated models for each level of complexity. Average frequency for the most common predictors was calculated as the average occurrence for each level of complexity. This approach compensates for the fact that the number of validated models increases dramatically with the numbers of predictors included. The leverage of individual predictors in each model was evaluated using the standardized coefficients for each model.

## Results

Three features displayed significant correlation with the outcome variable (MADRS): scaling exponent for full range (4 min–24 h; alpha full, negative correlation), and intradaily variability for 5 min and 30 min bins (IV5, IV30, positive correlation) (Fig. 2A). The brute force approach generated 14,892 models, out of which 3837 survived the filtering for internal validation. The average RMSE and *R*-squared in the models surviving internal validation were 1.84 ± 0.35, and 0.67 ± 0.11, respectively. The frequency of occurrence of individual predictors in models developed by brute force varies greatly across levels of complexity. However, alpha full, IV5 and IV30 display consistent frequencies across complexity levels (Fig. 2A).

Next, we evaluated the performance of models surviving internal validation in predicting the MADRS score in an independent population. The filtering criteria for external validation further reduced the number of validated models to 192 (Fig. 2B). The average RMSE and *R*-squared in the models surviving external validation were 2.70 ± 0.24, and 0.59 ± 0.09, respectively. In comparison with the simulated dataset, the average RMSE for validated models corresponds to models with a probability of occurrence below 0.001 (Fig. 2C). The analysis of standardized coefficients for the models surviving external validation revealed good consistency across models for most independent predictors (Fig. 2D). We further found that alpha full, IS5, and RA were included on average in >50% of the models (Fig. 2E).

**Fig. 3:**
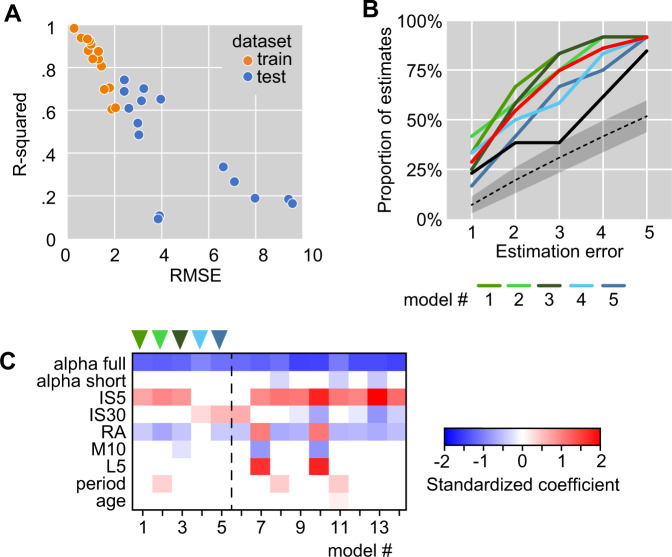
Development of multiple regression models using forward stepwise approach. **A** A total of 14 models forward stepwise multiple regression models survived external validation and were used for further analyses. The performance of selected models in predicting the depression score in the test dataset is displayed on the same axes for comparison with the performance on training dataset. **B** Evaluation of prediction of MADRS score at individual level for the selected models as compared to dummy model (constant output equal to the average of the sample; solid black line) and random prediction (dashed black line and shaded areas depict mean and 95% confidence intervals estimated over 1 million simulated datasets). The red line depicts the average performance of validated models developed by brute force. Line colors correspond to arrowhead colors in (**C**). **C** Leverage of individual predictors as illustrated by standardized coefficients. Models to the right of the vertical dotted line did not survive external validation. Models #1, 3, and 5 are further analyzed in Fig. 4.

These results indicate that depression severity scores correlate with features extracted by analyzing the pattern of activity recorded over several consecutive days. The brute force approach trains independent models and does not use information from previously generated models to increase the accuracy in subsequent steps. Therefore, we also used stepwise machine learning (ML) algorithms to train models of increasing complexity. This procedure yielded 18 models, which underwent internal and external validation as described above. Fourteen models survived the internal validation criteria and underwent external validation (Fig. 3A). Eight models had better accuracy than the dummy model, and 5 models survived filtering by external validation criteria (Pearson *R* > 0.576; RMSE < 3).

We addressed the distribution of estimation errors for all validated models and calculated the proportion of absolute residuals below 1 to 5 MADRS units in 1-unit increments (Fig. 3B). On average, the models surviving external validation criteria predict MADRS score within 2 units in 54% of the cases, within 3 units in 75% of the cases, and within 4 units in 87% of the cases. The distribution of estimation errors for the models trained by forward stepwise procedure was similar to the outcome of brute force approach (Fig. 3B). Next, we assessed the leverage of individual predictors in the models developed by forward stepwise procedure (Fig. 3C). The scaling exponent (alpha full) was present in all models surviving internal validation and had the largest absolute standardized score in all models, particularly in the models surviving external validation. In addition, interdaily stability calculated on 5- or 30-min bins (IS5, IS30) and the relative amplitude of circadian rhythms (RA) were included in the models surviving external validation.

**Fig. 4:**
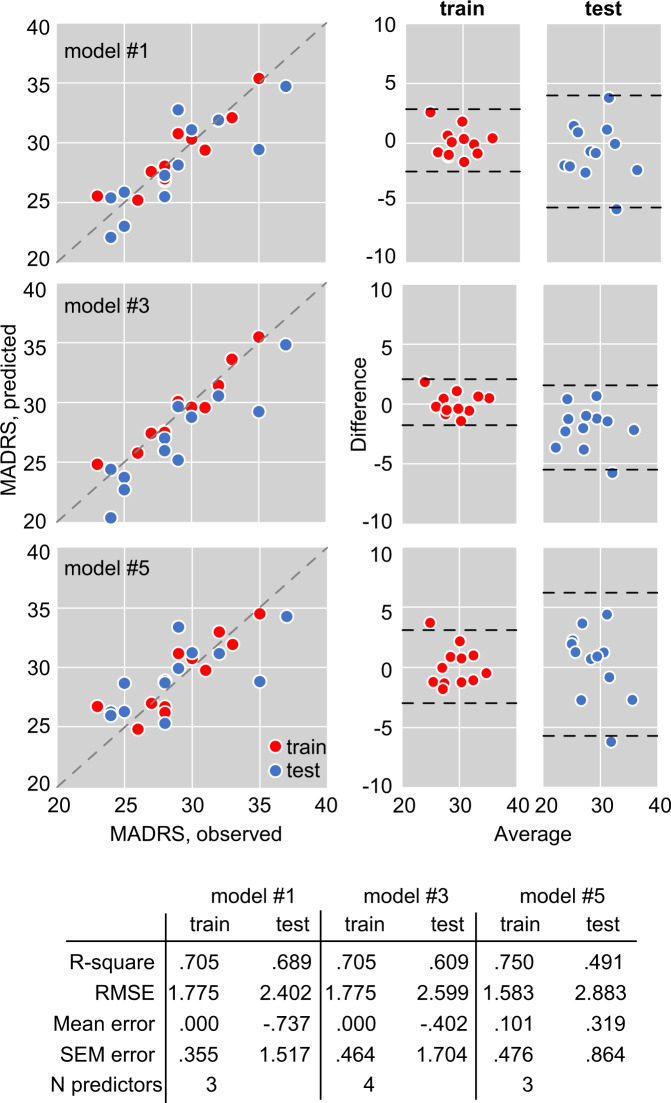
Performance of 3 selected models developed using stepwise approach. Left panels: predicted vs. observed MADRS. Dashed line depicts ideal correlation (identity between observed and predicted). Right panels: Bland–Altman plots for observed and predicted MADRS scores in train and test datasets to evaluate the agreement between observed and predicted values.

Lastly, we selected three models (best, intermediate, and worst performance in test dataset) for evaluation of agreement between observed and predicted MADRS scores (Fig. 4). The Bland–Altman plots indicate good agreement between observed and predicted score in both training and test datasets (Fig. 4).

## Discussion

Levels of activity and depression symptom severity are linked in a bidirectional manner: on average, higher severity correlates with lower levels of activity, particularly in the moderate activity band [14]; and increasing the levels of activity by physical exercise may in many cases reduce depressive symptoms [16, 18]. Here we show that symptoms of depression correlate with features of individual patterns of activity independently from actual activity levels. In addition, we bring proof-of-concept evidence that symptom severity can be predicted by analyzing the subject’s activity recorded over several consecutive days. We developed several multiple linear regression models which performed satisfactorily on a dataset independent from the training population. The models were generated using either a brute force approach or using forward-stepwise semi-supervised procedure. We also identified a number of features with frequent occurrence in the models surviving the external validation procedure. Our data supports the use of actigraphy recordings as minimally invasive objective measurement for the evaluation of depression patients.

Wrist actigraphy is a technique that allows long-term recording of activity with minimal discomfort and safety challenges for the subject. For major depressive disorder, the heterogenous pathological mechanisms leading to depressed mood as core symptom raise the challenge of finding objective behavioral features that correlate with symptom severity as measured by MADRS scale. The features we identified as particularly relevant estimate the complexity of activity patterns (scaling exponents); the strength of coupling between activity and circadian entrainers (interdaily stability, IS); and the robustness of circadian rhythms (RA). The coefficients point towards higher depression severity scores correlating with less complex patterns of activity, stronger coupling of activity with circadian entrainers, and less robust circadian rhythms. This is in line with earlier reports on less complex patterns of activity in patients suffering from depression [33], and with higher likelihood to get diagnosed with depression in subjects displaying blunted difference between day-time and night-time activity levels [1]. Our results also indicate that higher symptom severity is associated with higher IS, suggesting that stronger coupling with circadian entrainers is associated with more severe symptoms. Of note, stronger coupling with circadian entrainers can account for less complex patterns (estimated by scaling exponents), by reducing the contribution of high-frequency fluctuations. At the same time, stronger coupling with circadian entrainers does not warrant higher amplitude of circadian rhythms (estimated by RA), if the circadian fluctuations are of small amplitude (i.e., shorter, and less robust bouts of activity, as described previously [6, 10]). Taken together, our results point towards more severe symptoms being associated with decreased internal drive to steer one’s own activity in a circadian context, and instead passively follow circadian entrainers.

For internal validation of models, we used a heuristic approach considering the required accuracy of MADRS estimation. We selected RMSE as measure of accuracy because it penalizes all deviations and is sensitive to outlier values, and did not use average deviation, where the leverage of large positive and negative outliers can balance out and yield a misleading small average. Reports available in the literature estimate 95% CI = 7 face-to-face vs. telephone interview [34] and up to 2.75 units difference across testing occasions [35]. In our datasets, restricting to RMSE < 3 yielded an average accuracy of 2.7, and an absolute error below 3 in 75% of the cases. Another reference value we considered is the minimum clinically important difference for response to treatment, estimated to 2 units on MADRS scale [36]. Thus, the accuracy of the predicted score should be lower than 2 so that clinically relevant effects of antidepressant treatments are not obscured by estimation error. Models developed by brute force surviving external validation criteria approximate on average 54% of the case within 2 MADRS units, and in >90% of the cases in the best models. Larger datasets are required for further refining the model training approach, including internal validation prior to external validation on independent datasets.

Notably, the features we have extracted are sequence-dependent, and do not include summary statistics (e.g., total time inactive, or similar). In addition, our analyses consider the 24-h cycle as a continuum and do not ascertain crisp distinctions between active and resting intervals, nor do we implement any classification of samples or segments based on intensity of activity recorded. Therefore, our results offer a novel perspective, complementary to earlier reports on correlations between levels of activity and symptom severity [14, 15]. From a clinical perspective, our data may facilitate connections with molecular mechanisms behind onset of depressive episodes or the changes associated with response to treatment [26].

## Limitations of the study

All patients included in this study were diagnosed with a unipolar major depressive episode, and the model was trained to predict the MADRS score registered prior to the actigraphy recording, under the implicit assumption that activity was recorded in a stable state (i.e., no significant variations in symptom severity expected during recording time). Therefore, correlations between symptom severity and patterns of activity in other mood disorders (e.g., bipolar disorder or cyclothymia), or dynamic changes in response to treatment cannot be inferred from our results. In addition, correlations between patterns of activity and symptom severity in patients undergoing antidepressant treatment needs to be investigated separately. The impact of psychoactive drugs (acting on neurotransmitter systems which regulate circadian activity, e.g., glutamate, serotonin, noradrenaline, acetylcholine; reviewed in ref. [37]) on activity patterns of psychiatric patients is not fully understood [38].

A potential limitation of our study is the essential difference between inclusion criteria for train and test populations. Thus, the training dataset was collected form patients recruited for a clinical trial assessing the response to CBT. Implicitly, this leads to high variability in symptoms and pathological mechanisms. In contrast, the test dataset was acquired from patients included in the study only if they did not respond to SSRI drugs and would be eligible for ketamine treatment—hence a strong selection bias is expected. However, the fact that models trained on an intrinsically more variable population sample perform very well on the more strictly selected population support the applicability of our approach.

The availability of only two independent datasets with relatively low number of patients and narrow MADRS range further limits the extent of analyses due to the risk of overfitting the available pair of datasets. These limitations are particularly relevant when evaluating the models trained using the brute force approach. Nevertheless, the brute force approach highlighted the most frequently occurring features in externally validated models. The interpretation of the coefficients of individual predictors is consistent with clinical observations of alterations characteristic for depression. Further investigations focusing on increasing the number of recordings within study population, as well as on analyzing data from several independent populations are required for strengthening the biological significance of specific features.

Lastly, actigraphy data was collected using different devices between populations. This explains the significant between-group differences for features describing the magnitude of activity levels (M10; L5). This appears not to be a matter of concern, because these specific features are not identified as most relevant in the training dataset. This also implies that neither the actual peak of activity (i.e., most intense peak of activity during active phase), nor the trough of activity (typically associated with night-time activity or insomnia) are significant predictors of depression severity in our study populations. Interestingly, neither are the locations of the circadian peak and trough significant predictors for symptom severity. In contrast, these features appear relevant for distinguishing between healthy control and MDD patients [8, 12]. These data suggest that while the magnitude and location of circadian peaks and troughs of activity may have diagnostic value, they do not correlate with symptom severity.

From a clinical application perspective, our results indicate that actigraphy could be a useful tool in the individual evaluation of patients with depression. Larger confirmatory studies are needed before clinical implementation.

## Data Availability

The Matlab code used for feature extraction and model training is available upon request.
